# Leaf surfaces and neolithization - the case of *Arundo donax L*

**DOI:** 10.3389/fpls.2022.999252

**Published:** 2022-10-05

**Authors:** Sílvia C. Nunes, Ana P. Gomes, Paulo Nunes, Mariana Fernandes, Ana Maia, Eunice Bacelar, João Rocha, Rebeca Cruz, Aline Boatto, Ajith P. Ravishankar, Susana Casal, Srinivasan Anand, Verónica de Zea Bermudez, António L. Crespí

**Affiliations:** ^1^ Fib EnTech - Fiber Materials and Environmental Technologies, University of Beira Interior, Covilhã, Portugal; ^2^ Centro de Química Vila Real (CQ-VR), University of Trás-os-Montes e Alto Douro, Vila Real, Portugal; ^3^ Department of Chemistry, University of Trás-os-Montes e Alto Douro, Vila Real, Portugal; ^4^ Centre for the Research and Technology of Agro-Environmental and Biological Sciences (CITAB), Department of Biology and Environment, University of Trás-os-Montes e Alto Douro, Vila Real, Portugal; ^5^ Herbarium and Botanical Garden, University of Trás-os-Montes e Alto Douro, Vila Real, Portugal; ^6^ Associated Laboratory for Green Chemistry (LAQV) of the Network of Chemistry and Technology (REQUIMTE), Department of Chemical Sciences, Faculty of Pharmacy, Laboratory of Bromatology and Hydrology, University of Porto, Porto, Portugal; ^7^ Department of Applied Physics, School of Engineering Sciences, KTH Royal Institute of Technology, Albanova University Centre, Stockholm, Sweden

**Keywords:** *A. donax* L. leaf, environmental adaptation, wax composition, hydrophobicity, optical properties, neolithization, chemophenetics and inorganic composition

## Abstract

*Arundo donax L*. (Arundinoideae subfamily, Poaceae family) is a sub-tropical and temperate climate reed that grows in arid and semi-arid environmental conditions, from eastern China to the Mediterranean basin, suggesting potential adaptations at the epicuticular level. A thorough physical-chemical examination of the adaxial and abaxial surfaces of *A. donax* leaf was performed herein in an attempt to track such chemophenetic adaptations. This sort of approach is of the utmost importance for the current debate about the hypothetical invasiveness of this species in the Mediterranean basin versus its natural colonization along the Plio-Pleistocene period. We concluded that the leaf surfaces contain, apart from stomata, prickles, and long, thin trichomes, and silicon-rich tetralobate phytolits. Chemically, the dominating elements in the leaf ashes are oxygen and potassium; minor amounts of calcium, silicon, magnesium, phosphorous, sulphur, and chlorine were also detected. In both surfaces the epicuticular waxes (whose density is higher in the adaxial surface than in the abaxial surface) form randomly orientated platelets, with irregular shape and variable size, and aggregated rodlets with variable diameter around the stomata. In the case of green mature leaves, the dominating organic compounds of the epicuticular waxes of both surfaces are triterpenoids. Both surfaces feature identical hydrophobic behaviour, and exhibit the same total transmittance, total reflectance, and absorption of incident light. The above findings suggest easy growth of the plant, remarkable epidermic robustness of the leaf, and control of water loss. These chemophenetic characteristics and human influence support a neolithization process of this species along the Mediterranean basin.

## 1 Introduction

*A. donax*, commonly known as giant reed (Jiménez-Ruiz et al., 2021), is a tall, perennial, C3 grass species, sub-tropical and temperate climate cane. Included in the *Arundinoideae* subfamily of the *Poaceae* family, it grows in a wide variety of soil types, ranging from coarse sands to heavy clays, but generally prefers well-drained soils above the mean water level in freshwater streams (Ceotto and Candilo, 2010).

**Figure 1:**
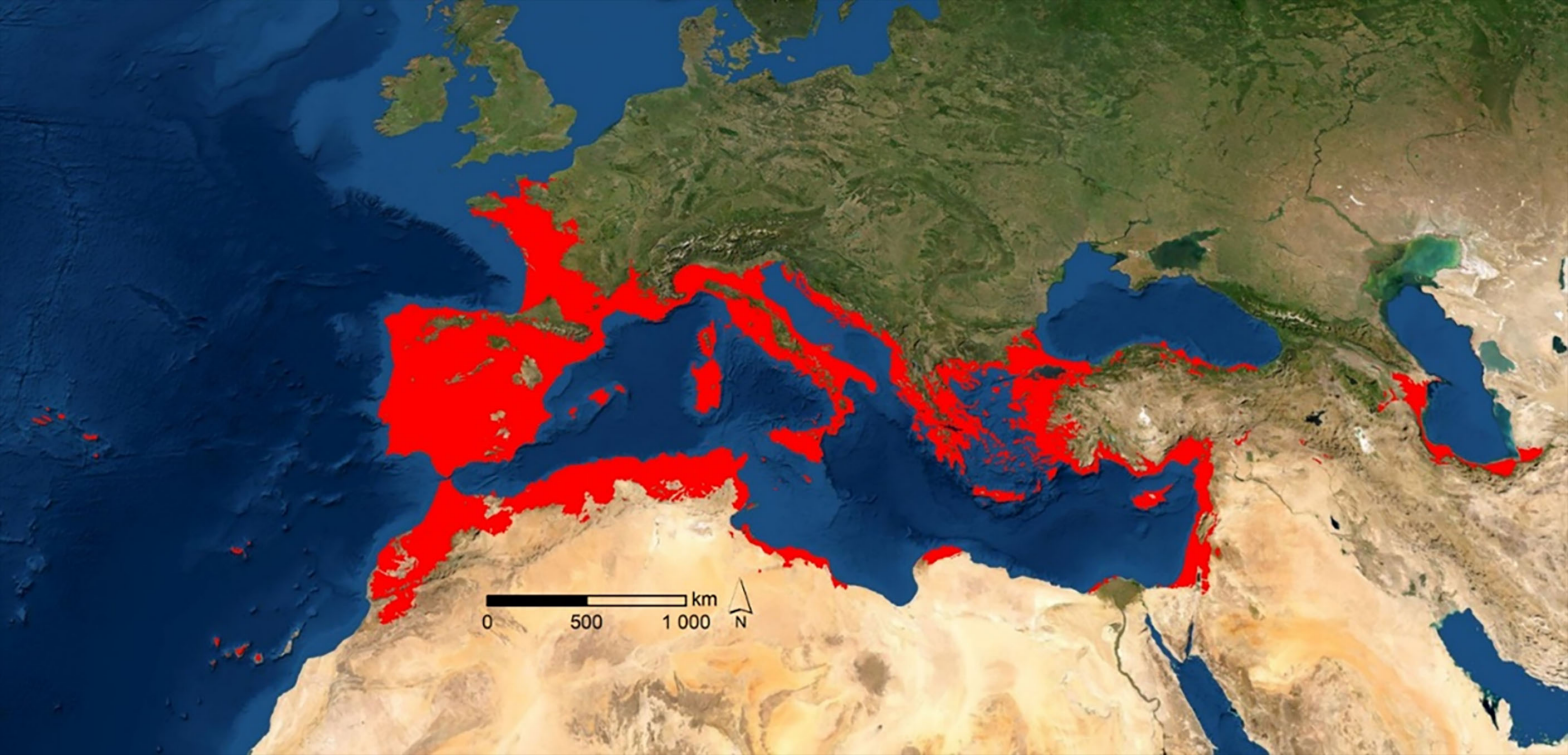
Potential natural habitat distribution of A. donax (red areas represent ≥ 50% of probability of occurrence).

A genetic approach based on plastid DNA placed the origin of *A. donax* on the southern Caspian Sea, southern Iran, and the Indus valley (Hardion et al., 2014a). Reproductive abilities, as well as very efficient responses of this species to environmental alterations under riparian conditions, provided *A. donax* with a great capacity to dominate large extensions across this habitat (red areas in **Figure 1**). The ability to quickly colonize riparian habitats was the main argument to include *A. donax* among the allochthonous invader species of the Mediterranean basin and the occident of Eurasia (Jiménez-Ruiz et al., 2021). These ecological abilities have been very useful for ornamental purposes or, more recently, for bioenergetic uses (Corno et al., 2014; Webster et al., 2016; Margit, 2017; Antal, 2018). The taxonomic and morphogenetic diversity in eastern Asia is, however, greater than along the Mediterranean basin (Danin, 2004; Mariani et al., 2010; Hardion et al., 2012; Hardion et al., 2014a; Hardion et al., 2014b), an evidence that reveals apparent genetic homogeneity in the latter region, with haplotypic hybridization between two different morphogenomes (henceforth designated as T1 and T2). Notwithstanding, another issue could be brought to the discussion. A biogeographic perspective of recent natural colonization, without human interaction, could be also involved. Indeed, migrations inside and across the Mediterranean basin were very frequent in the Plio-Pleistocene glacial-interglacial events (Fiz-Palacios et al., 2010; Carlson et al., 2012; Nieto Feliner, 2014; Martín-Hernanz et al., 2021). Unravelling a controversy such as this demands as much information as possible, as well as different analytical approaches.

Herein we propose that a physical-chemical analysis of the adaxial and abaxial surfaces of the *A. donax* leaf, focused on its potential distribution, environmental behavior, morpho-anatomy and histology, microstructure, chemical organic and inorganic composition, wettability properties, and optical features, is a valuable strategy to get rich additional insight into the reasons behind the presence of this species in the Mediterranean flora. Our main motivation to carry out the present work was the fact that the leaf waxes were found to reveal important information regarding the ecological strategies of plants from a chemophenetics standpoint (Janaćković et al., 2019; Rajčević et al., 2020; Marques et al., 2021).

## 2 Materials and methods

### 2.1 Potential distribution

The potential area of distribution of *A. donax*, shown in **Figure 1**, was calculated on the basis of the polyploidy supported by haplotypes T1 and T2, and their proposed hybridization along the Mediterranean region and the occident of Eurasia (Hardion et al., 2014b). The output derived from Maxent modelling (in ASCII format) was input into ArcGIS software version 9.2 (ESRI, Redlands, California, USA) as floating-point grids (Pearson et al., 2007), and the occurrence probability of the species at each site was mapped. The areas with a potential habitat suitability over 50% were selected. Using Raster to Point tool, potential habitat area maps were then transformed into point features. We note that the potential distribution was always confirmed in herbaria or publications. In addition, the existence of isolated references, outside the potential area obtained, was also checked. Because of their low probability of occurrence, the potential map deduced does not include these locations. With the Multi Values to Point extraction tool, and with the climate data from worldclim.org, altitudinal and thermopluviometric data were assigned to each point.

### 2.2 Environmental characterization

The environmental characterization, which relied on the numerical matrix obtained above, was performed using the Pearson correlation matrix and the Principal Component Analysis (PCA) on the variables, and the individuals, thus comprising the two haplotypes. The as-derived information was represented by dispersal graphics for the first three factors, in order to describe more than 80% of the information contained in the correlation matrix.

Classification approaches were also employed to perform the environmental characterization without any previous correlation. In this context a Stepwise Discriminant Canonical Analysis (DCA) was used to show environmental differences along the biogeographic transept of interest (from eastern Eurasia to the Mediterranean basin and southwestern Europe). Within this sort of classification analysis, each environmental variable (independent variable) is tested, and its significance is measured by the Willks´ lambda statistic with an F-test and significance 0, using SPSS software version 27.0 (IBM Corporation, New York, USA).

### 2.3 Leaf collection

The *A. donax* leaves were collected during the spring and summer 2021, at the gardens of the Campus of the Faculty of Health Sciences of the University of Beira Interior, Covilhã, Portugal. All the samples were collected from adult plants during daytime, under sunny conditions, and at temperatures ranging between 18 and 30°C. Samples were classified as juvenile, green mature, and senescent, depending on their growth stage.

#### 2.3.1. Leaf calcination

For the analysis of the inorganic components, the collected plant samples were washed with deionized water. Afterwards, the samples were dried in an electric oven at 105°C for 24 h. In order to remove the organic matter, the samples dried at 105°C were then cut into small pieces and transferred to porcelain crucibles. The crucibles were incinerated at 600°C/700°C for 6 h (Carnelli et al., 2001; Payá et al., 2018) or 800°C for a period of 2 h, at a heating rate of 10°C min^-1^ (Villar-Cociña et al., 2018) in a muffle furnace. One part of the ash contents obtained at 600°C was transferred to test tubes and hydrogen peroxide (H_2_O_2_, Fischer Scientific, 30%) was added to submerge the ash. Then the test tubes were transferred to a water bath at 80°C for 1 h. The supernatant was decanted, and the ash rinsed twice in distilled water. Hydrochloric acid (HCl, Fischer Scientific, 10%) was added and the mixture was subsequently incubated again at 80°C for 1 h. Afterwards the mixture was decanted off, washed twice in distilled water, and centrifuged for 15 min at 7500 r.p.m. The supernatant was decanted off and the pellet was washed twice in distilled water. Finally, the ash was dried for 24 h at 60°C. All the ashes obtained using different procedures were analyzed by scanning electron microscopy and X-ray diffraction.

### 2.4 Morpho-anatomical and hystological characterization

Green mature leaf samples for light microscopy examination were collected and immediately after fixed in a formalin:acetic acid:70% ethanol (FAA) solution (volume ratio 1:1:18) for 24 h. Subsequently, the samples were dehydrated in ethylic series and included in paraffin. The as-derived blocks were sectioned (5 micro m thick) through a rotative microtome (Leica RM 2135; Leica Microsystems, Nussloch, Germany) and stained with 1% toluidine blue O (Merck KGaA, Darmstadt, Germany) in 0.1 M phosphate buffer (pH 6.8) for 10 min using the method of O’Brien and McCully (O’brien and McCully, 1981). Sections were mounted with Entellan (Merck, Darmstadt, Germany). Transverse sections of the midrib at median level, as well as transverse sections of the leaf-blade region and in the margin, were obtained and examined under a light microscope (Olympus IX51, Olympus BioSystems, Munich, Germany), and photographed with a digital camera (ColorView III; Soft Imaging System GmbH, Münster, Germany). The images were analysed with the Olympus software Cell. To assess the histological differences between green mature and senescent leaves, fresh free-hand sections were also performed using a hand microtome and a straight razor blade. The cuttings were cleared with sodium hypochlorite (commercial grade) and stained with Sudan III (Merck KGaA, Darmstadt, Germany) which stains lipidic compounds in a deep red color to highlight the cuticle (Bacelar et al., 2004). Measurements of total leaf thickness were performed on the cross-sections of six green mature leaves and six senescent leaves with Digimizer Image Analysis software (MedCalc Software, Belgium).

### 2.5 Structural characterization

#### 2.5.1 Variable pressure scanning electron microscopy (VP-SEM) and energy dispersive X-ray spectroscopy (EDS)

The hierarchical structure of the adaxial and abaxial surfaces of a green mature *A. donax* fresh leaf and the dried leaf were examined by VP-SEM acquired at 20 kV on a Hitachi S-3400N type II microscope. The sample was mounted on appropriate stubs, using double-stick adhesive, electrically conductive carbon tape. Elemental mapping analysis on microscopic sections of the *A. donax *L. leaf fresh and the elemental analysis of the dried leaf were performed by EDS. The acquisition time was 50 s. Maps of the distribution of each element across the same area were produce, allowing show the spatial distribution of elements in a sample.

#### 2.5.2 Scanning electron microscopy (SEM)

SEM images of the cuticular wax layers of the adaxial and abaxial surfaces of a green mature *A. donax* leaf were obtained on a Hitachi S-3400N type II microscope (Hitachi, Chiyoda, Japan) at high vacuum using a voltage of 20 kV. Elemental analysis of the ashes obtained during the calcination without any coating were performed by EDS, using the previous conditions. Prior to SEM analysis, a piece of the *A. donax* L. fresh leaf was fixed with 2.5% (v/v) glutaraldehyde, kept overnight in a refrigerator at 4°C, and subsequently processed through immersion for 7 min each in a series of increasing concentrations of ethanol (10, 20, 30, 50, 70 and 100%). To prevent leaf damage during air drying, the critical point drying (CPD) technique (Emitech, K850 critical-point dryer, France) was employed to replace all of the ethanol with liquid carbon dioxide (CO_2_) under pressure. Using this procedure, the surface structure of a specimen was preserved which could otherwise be damaged due to surface tension when changing from the liquid to gaseous states. The volume of liquid CO_2_ was replaced several times until ethanol was no longer present in the purge line. Once the dried material was removed, it was stored in a desiccated environment until further examination. Finally, the sample was coated with gold.

#### 2.5.3 Polarized optical microscopy (POM)

POM images were taken using an OPTIKA B-600POL microscope equipped with an 8 M pixel Digital Photo Camera and analyzed using the OPTIKA Vision Pro software.

#### 2.5.4. X-ray diffraction (XRD)

A Rigaku DMAX III/C diffractometer was used to make a 3-70° (2θ) scan with the reflection method (operation voltage of 30 kV and a current of 15 mA). The ash sample was deposited on a glass substrate.

#### 2.5.5. Fourier-transform infrared (FT-IR) spectroscopy

The FT-IR spectra were collected on a Thermoscientific Nicolet iS10: smart iTR in the 4000–500 cm^−1^ range by averaging 128 scans at a resolution of 1 cm^−1^. The sample (2 mg) was finely ground and mixed with ~ 175 mg of dried potassium bromide (KBr, Merck, spectroscopic grade) and pressed into pellets. Prior to recording the spectra, the pellet was stored under vacuum for 1 week at room temperature to reduce the levels of adsorbed water.

### 2.6 Chemical characterization

#### 2.6.1. Epicuticular and intracuticular waxes extraction

To extract independently the epicuticular and intracuticular waxes of the adaxial and abaxial surfaces of a green mature *A. donax* leaf, the freeze-embedding (cryo-adhesion) method described in detail elsewhere (Ensikat et al., 2000) was adopted. Fresh leaf samples of approximately 2 × 2 cm^2^ were gently cut. To isolate the epicuticular wax, a drop of glycerol (Sigma-Aldrich, 99.5%) was applied on the surface of a metal plate to form a single drop. Then a piece of a leaf was placed on the drop, pressed gently, and the resulting set was submerged slowly into liquid nitrogen (N_2_) for 10 s. Then the leaf sample was lifted off the frozen glycerol drop in which the epicuticular wax remained embedded. The epicuticular wax layer adhered to the plate was removed with chloroform (Honeywell, Riedel-de-Haën, ≥99.8%) and transferred to glass tubes. After the solvent was evaporated, the glass tubes were sealed. To remove the intracuticular wax, the above process was repeated using the same leaf sample. The glycerol drops were placed exactly in the same spots considered in the isolation of the epicuticular wax. The residues of both types of waxes were subject to independent chemical characterization.

#### 2.6.2. Chemical and statistical characterization of the epicuticular and intracuticular waxes

After cryo-adhesion, the internal standard (tetradecane, 50 μL, 500 ng mL^-1^) was added to cuticular residues that were then redissolved in chloroform. Derivatization, chromatographic separation and detection was performed as described in a previous study (Rodríguez et al., 2021). Results are expressed as a relative response of the total ion chromatographic areas after normalization to the internal standard. Extractables and leachable-screening standard for gas chromatography (GC) (ELSS, TraceCERT^®^, Sigma-Aldrich, USA) were used to detect volatile and some semi volatile compounds from packaging materials and tubing that may interfere with the analysis. Results are expressed as average values and standard deviation from duplicate analysis of each sample. Statistical analyses were performed at a 5% significance level, using SPSS software (version 27.0, IBM Corporation, New York, USA) as detailed by Rodríguez et al. (Rodríguez et al., 2021). In brief, normal distribution of the residuals and the homogeneity of variances were evaluated through the Shapiro−Wilk test and the Levene test, respectively. Then, all dependent variables were studied using a one-way analysis of variance (ANOVA), subjected or not to Welch correction, followed by suitable *post-hoc* tests. For principal component analysis (PCA), data were previously standardized and oblimin with Kaiser normalization was selected as the rotation method considering that dependent variables were highly correlated.

### 2.7 Thermal characterization

Differential scanning calorimetry (DSC) thermograms were recorded using a differential scanning calorimeter (Netzsch DSC 204, Germany). The wax sample with approximately 3-4 mg was placed in a hermetic sealed aluminum pan with a pinhole. The sample was heated from 30 to 100 °C at a heating rate of 5 °C min^-1^, followed by cooling from 100°C back to 30 °C at the same rate. The purge gas used was N_2_.

### 2.8 Wettability characterization

The wettability of the adaxial and abaxial surfaces of juvenile and green mature *A. donax* leaves were assessed by means of static water contact angle (WCA) measurements using the sessile drop method. The WCA gives an inverse measurement of the adhesion between water and a solid. For WCA > 90° the surface is said to exhibit hydrophobicity. If the WCA > 110°, the surface may be classified as non-wettable. A surface with WCA > 130° is named highly non-wettable. At last for WCA > 150° the surface is usually addressed as superhydrophobic (Wang et al., 2015). The WCAs were measured in a temperature-controlled chamber at 26 ± 1 °C with ultra-pure distilled water using a Krüss DSA25S drop shape analyser equipped with a tilting table and controlled by the software ADVANCE. The volume of the water droplets was kept constant at 5 μL. WCAs were measured from digital images acquired by a video camera using the Young-Laplace fitting. The WCA values were measured at least 5 different spots and at each spot 5 measurements were performed (**Figure S1** of the **Supplementary Material**). The results reported correspond to the average value of the measurements performed at each spot. The error analysis of the data was implemented by arithmetic mean of the root mean square error.

### 2.9 Optical characterization

#### 2.9.1 Commission Internationale d’Éclairage (CIE) L*a*b* color coordinates

The CIE L*a*b* color coordinates were measured with a Chroma Meter CR-300 Minolta (Osaka, Japan). The L*, a*and b* parameters (where L* is the lightness, a* represents the green–red component, and b* represents the blue–yellow component) of the adaxial and abaxial surfaces of juvenile, green mature, and senescent *A. donax* leaves were calculated from the average values obtained from 3 measurements on each surface. During the measurement, the equipment was placed perpendicularly to the flat surface of the leaf.

#### 2.9.2 Optical transmittance and reflectance

The total and diffused reflectance, total transmittance, and absorbance of light on the adaxial and abaxial surfaces of a green mature *A. donax* leaf were measured using a Perkin-Elmer Lambda 900 spectrophotometer in combination with a 150 mm integrating sphere. Details of the experimental setup employed in the experiments were described in detail elsewhere (Rodríguez et al., 2021). Angle resolved measurements were also performed. To measure angle-dependent absorption, the leaf was mounted on a rotating stage and placed at the center of the integrating sphere. The total light inside the integrating sphere is a contribution from both reflectance and transmittance (R+T). In this way, the total absorbance is calculated as 100-(R+T) in %.

## 3 Results and discussion

### 3.1 Environmental characterization

The environmental characterization carried out revealed marked differences between the western and eastern haplotypes T1 and T2, respectively, and the polyploidy differentiation observed for Mediterranean *A. donax* (Hardion et al., 2014a).

**Figure 2:**
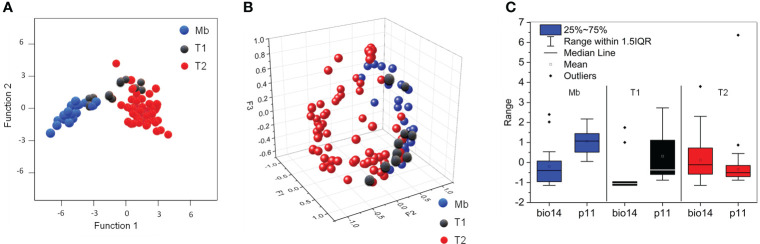
**(A)** DCA for the strategies of *A donax*: a biogeographic strategy (Mb, blue symbols) and two haplotypic strategies (T1, black symbols, and T2, red symbols); **(B)** PCA for the first three factors (F1-F3) confirming the DCA results *via* correlation between climatic variability per site, and biogeographic and haplotypic strategy; **(C)** Box-plot for the most discriminant climatic variables (bio14 and p11) per biogeographic and haplotypic strategy.

The DCA derived for these three strategies (one biogeographic and two haplotypic) is represented in **Figure 2A**. The average precipitation in November (p11) and the precipitation of the driest month (bio14) are the most discriminant environmental variables (**Table S1** in the **Supplementary Material**). The intermediate climatic conditions of T1, between T2 and the specimens located along the Mediterranean basin (Mb), were confirmed by the PCA performed for the first three factors F1, F2 and F3 (89.71% variance analysed from the Pearson correlation matrix) (**Figure 2B**).

The differences are essentially the monthly average maximum and minimum temperatures (tM and tn, respectively), as deduced from the PCA performed for Pearson correlation matrix. The ranges for p11 and bio14 reveal clear differences of the average precipitation in November for Mb and T2, and intermediate values for T1 (**Figure 2C**). We may infer from these data that the presence of *A. donax* across the Mediterranean basin describes a continuous climatic response along the eastern and western of Eurasia, and northern Africa.

### 3.2 Morpho-anatomy and histology

**Figure 3:**
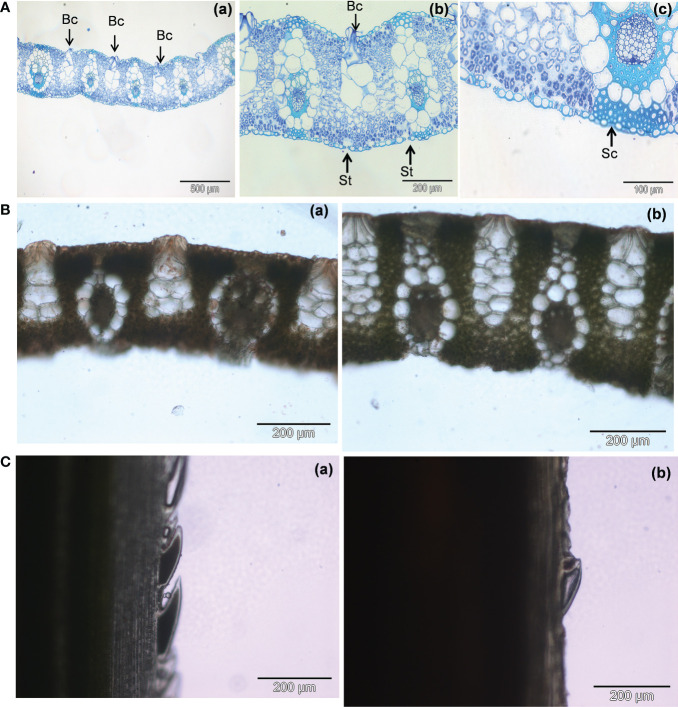
**(A)** Light micrographs of cross-sections of a green mature *A donax* leaf stained with toluidine blue: **(a)** Overview of leaf blade; **(b)** Stomata in the abaxial surface; **(c)** Bands of sclerenchyma tissue associated with the vascular bundles. Bc- bulliform cells; St – stomata; Sc - sclerenchyma. **(B)**: Light micrographs of cross-sections of an *A donax* leaf stained with Sudan III: **(a)** green mature leaf, **(b)** senescent leaf. **(C)**: Differences in leaf blade margin of fresh leaf cross sections of *A donax* leaves: **(a)** green mature leaf, **(b)** senescent leaf.

The cross-sections of a green mature *A. donax* leaf blades show a typical monocot organization (**Figure 3Aa**). The epidermis of this leaf is the usual one for grasses (Sylvester et al., 2001; Smith, 2005). The mesophyll is undifferentiated, and all the veins appear parallel to each other (Monteiro et al., 2015). Bulliform cell rows can be distinguished in the intercostal zone of the adaxial surface (**Figures 3Aa, b**). Motor or bulliform cells are larger and extend further into the mesophyll.

The walls are thick, and the cells are hyaline and without chlorophyll that distinguishes it from other epidermal cells. Stomata are seen in the intercostal zone (**Figure 3Ab**). Their concentration is higher in the abaxial surface than in the adaxial surface, in agreement with previous reports by Khan et al. (Khan et al., 2017). The vascular bundles are surrounded by bundles sheaths and a continuous band of sclerenchyma throughout the epidermis (**Figure 3Ac**). The analysis of the fresh free-hand sections stained with Sudan III allowed us calculating total leaf thickness differences between the green mature (289.2 ± 14.6 µm) (**Figure 3Ba**) and senescent (411.8 ± 13.7) (**Figure 3Bb**) leaves.

The presence of a cuticular wax coating is more evident in the green mature leaves (cf. red stain on the top surface of the leaf cross-section in **Figure 3Ba**). The higher concentration of epicuticular waxes in the younger leaves may be related with protection purposes, and also with higher reflectance and resistance to drought (Shelton, 2012; Zhu and Xiong, 2013). Interestingly, the leaf margins of the *A. donax* leaf are rough to the touch (Ditomaso and Healy, 2003). We observed that the sharp pointed segments detected in the green mature leaves (**Figure 3Ca**) became worn with time in senescent leaves (**Figure 3Cb**).

### 3.3 Microstructure

**Figure 4:**
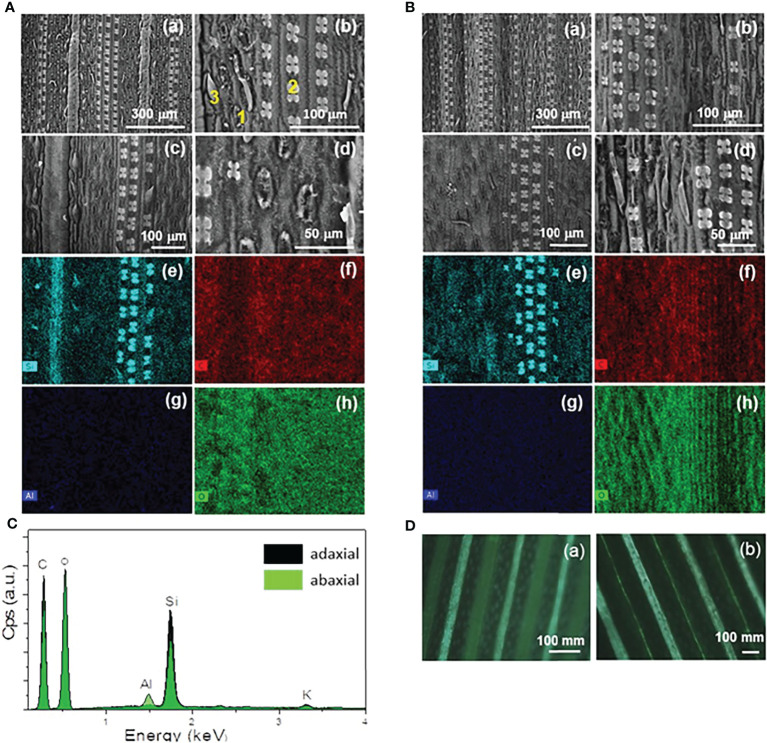
VP-SEM images **(a-d)** of the adaxial **(A)** and abaxial **(B)** surfaces of a green mature *A donax* leaf. EDS mapping images of VP-SEM image **(c)** for **(e)** silicon (Si), **(f)** carbon **(C)**, **(g)** aluminum (Al), and **(h)** oxygen (O). EDS spectra **(C)** of the adaxial (dark green area) and abaxial (light green area) surfaces corresponding to the entire area shown in the VP-SEM images of A(c) and B(c), respectively. **(D)** POM images (crossed polarizers) of the adaxial **(a)** and abaxial **(b)** surfaces.

The adaxial and abaxial surfaces of green mature *A. donax* leaves were analyzed by VP-SEM (**Figures 4A, B, a–d** respectively) and EDS (**Figures 4A, B, e–h**, respectively). **Figure 4C** shows the EDS spectra obtained for both surface for the five elements analyzed, i.e., carbon (C), oxygen (O), aluminum (Al), potassium (K) (not shown), and silicon (Si).

The VP-SEM images reveal numerous stomata (1) prickles (2) and long-thin hairs (3) (i.e., trichomes) in both surfaces. The tetralobate phytolits also detected (**Figures 4A, B, b–d**), are rich in Si, as confirmed by EDS (**Figures 4Ae, Be**). They are three-dimensional (3D) structures accumulated in the amorphous form. We recall that the amount of silica in a plant is controlled by: (1) extrinsic factors, such as ambient temperature, humidity, soil type, and soil moisture; and (2) intrinsic factors, such as the phenological state of plant (Fernández Honaine and Osterrieth, 2012; Pepim.G. et al., 2012; Shakoor et al., 2016). The aligned patterns and regular spacings of both surfaces of the leaf are clearly evident in the POM images represented in **Figure 4Da, b**).

**Figure 5:**
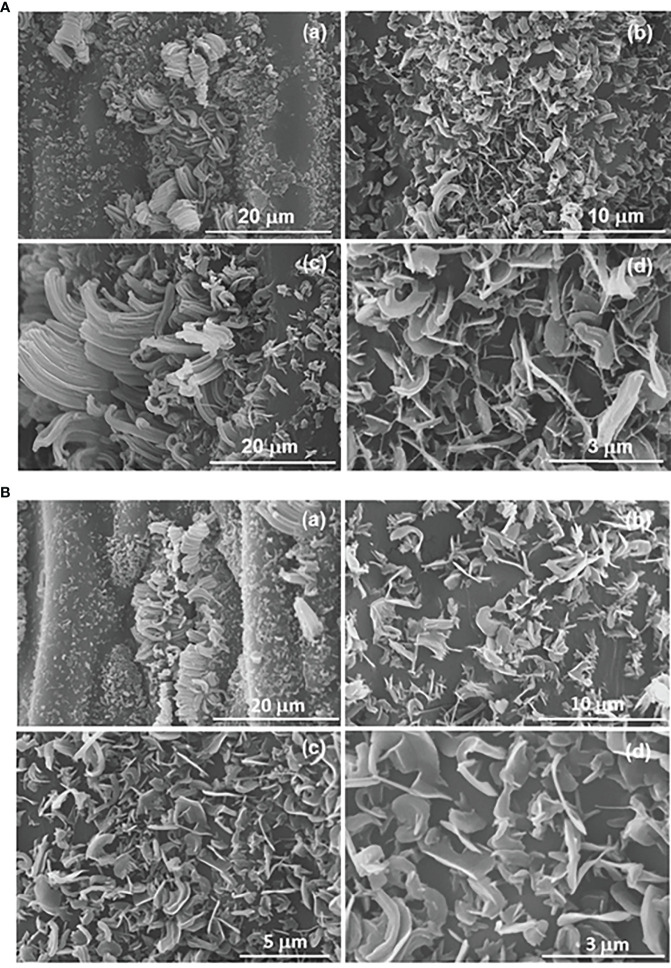
SEM images of the astomatous adaxial **(A)** and stomatous abaxial **(B)** surfaces of a green mature A donax leaf, and their randomly orientated platelets of epicuticular wax. The small letters are to differentiate the various images when referring in the text.

The micromorphology of the epicuticular wax on the astomatous adaxial and stomatous abaxial surfaces are represented in the SEM images of **Figures 5A, B**, respectively. In both surfaces the epicuticular waxes emerge as essentially randomly orientated platelets (**Figures 5A, b–d** and **Figures 5A, b–d**), with irregular shape and large variation in size (Barthlott et al., 2017). In addition, aggregated rodlets with variable diameter along the length of their axis are discerned around the stomata (see arrows in **Figures 5A, a–c, Ba**). Comparison of **Figures 5Ab, Bb** allows concluding that the epicuticular wax density is markedly higher in the adaxial surface.

### 3.4 Chemical composition

To track the inorganic components, present in the *A. donax* leaf, samples were first calcinated to eliminate the organic matter. EDS and XRD analyses were subsequently performed.

The elemental composition deduced from the EDS data for A. *donax* fresh leaves, for leaves dried at 105 and 600 °C, for the ashes produced at two different calcination temperatures (600 and 800 °C), and for ashes formed after chemical treatment of the ash resulting from calcination at 600 °C, are summarized in **Table 1**.

**Table 1:** Results of the element analysis (wt. %) of fresh, dried, and calcinated *A. donax* leaves (mean and standard deviations).

Element	Leaf				Ash			
	Treatment							
	No		105 °C	600 °C	600 °C, 6 h	600 °C, 6 h, H_2_O_2_, HCl	800 °C, 2 h	
	adaxial	abaxial	adaxial	adaxial			
C	43.26± 9.85	41.43± 9.39	40.85± 9.31	6.24± 2.74	5.27± 2.01	2.69 ±1.41	1.33± 1.14
O	50.83± 11.47	52.39± 11.73	52.55± 11.77	45.15± 12.25	45.34± 11.09	48.90± 11.96	38.34± 11.06
Si	5.26± 0.50	4.93± 0.47	4.91± 0.47	9.26± 0.88	8.95± 0.81	16.62± 1.43	9.39± 0.87
Al	0.16± 0.07	1.06± 0.15	1.05± 0.15	–	–	–	–
K	0.50± 0.08	0.40± 0.08	0.39± 0.08	23.55± 1.54	24.58± 1.54	3.26± 0.26	24.00± 1.55
Mg	–	–	–	1.46± 0.23	1.20± 0.19	5.87± 0.69	2.89± 0.39
P	–	–	–	1.18± 0.16	1.18± 0.15	3.64± 0.34	3.54± 0.35
S	–	–	0.11± 0.06	3.51± 0.32	3.47± 0.30	0.38± 0.09	4.13± 0.37
Cl	–	–	–	5.92± 0.47	6.20± 0.47	0.59± 0.10	7.60± 0.59
Ca	–	–	0.14± 0.06	3.74± 0.30	3.81± 0.28	17.78± 1.07	8.58± 0.59
Na	–	–	–	–	–	0.26± 0.10	0.28± 0.11

The *A. donax* leaf dried at 105 °C presents the same five constituents detected in the fresh leaf, with practically the same relative proportion (**Table 1**), plus trace amounts of sulfur (S) (0.11%) and calcium (Ca) (0.14%). The ashes obtained through the calcination of leaves at 600 and 800 °C are essentially formed of O, Si, K, chlorine (Cl), and S, plus trace amounts of magnesium (Mg) and phosphorus (P) (**Table 1**). None of the ashes contains Al. Apart from O, the second most abundant element in all ashes is K (ca. 24%), indicating the alkaline nature of the samples. The elements detected in the *A. donax* ashes in this work are practically the same as those reported by other authors, although their *A. donax* leaf ash contained a small amount of Al (Payá et al., 2018), but no sodium (Na).

**Figure 6:**
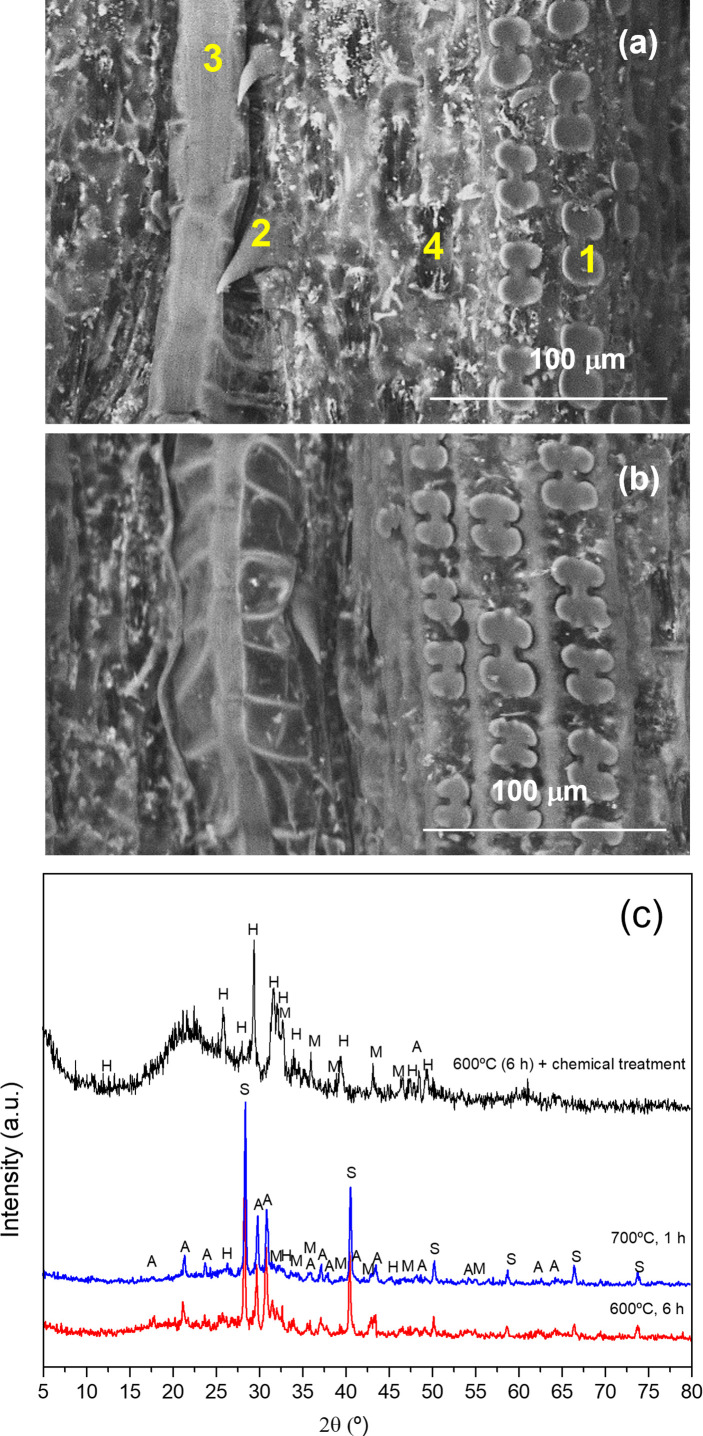
| VP-SEM images of the adaxial **(a)** and abaxial **(b)** surfaces of the *A donax* leaf after calcination at 600 °C for 6 h. 1: phytolith; 2: trichome; 3: nerve; 4: stomata. XRD patterns of the ash obtained from the *A donax* leaf calcinated at 600 °C for 6 h (red line), 700 °C for 1 h (blue line), and 600 °C for 6 h, followed by chemical treatment) (black line). S: sylvite; A: arcanite; M: magnesite; H: hidroxylapatite) **(c)**.

The EDS elemental analysis of the cellules of the adaxial surface of the *A. donax* leaf (**Figure 6**) performed after calcination at 600°C allowed inferring that the Si (wt. %) content varied significantly with the type of cellule, the highest amount being observed in the phytoliths (28.65%) (**Table 2**). However, elemental analysis of the phytoliths (1 in **Figure 6a**) also revealed the presence of other elements besides Si, namely Mg, P, S, Cl, and Ca, in small amounts, and K and O in higher amounts (**Table 2**). These elements are present in the cytoplasm of the host cells and were observed only after calcination solely or calcination plus chemical treatment. The same elements were also identified in the other cellules, but with variable amounts (**Table 2**, 2-4 in **Figure 6a**). In the nerves and the stomata Na was also detected in small quantities (0.1-0.2%). Shakoor et al. (Shakoor et al., 2016) examined phytoliths of the *A. donax* leaf, and reported the same elements detected herein, except S and P. In addition, these authors found nitrogen (N) and Al (**Table 2**). In the study of Payá et al. (Payá et al., 2018) the phytoliths appeared to contain only six elements (**Table 2**).

**Table 2:** EDS element analysis data (wt. %) of the ash produced by calcination at 600 °C for 6 h of special cellular tissues of the adaxial surface of the *A. donax* leaf (mean and standard deviations).

Element	Phytolith			prickle hair	Nerves	Stomata
	This work	(Shakoor et al., 2016)	(Payá et al., 2018)			
C	1.85 ± 1.04	25.30 ± 1.06	–	3.53 ± 1.38	2.46 ± 1.30	2.45 ± 1.43
O	49.19 ± 10.58	32.25 ± 2.15	58.31	57.76 ± 10.58	48.60 ± 11.55	41.94 ± 11.54
Na	–	–	–	–	0.11 ± 0.07	0.28 ± 0.11
Mg	0.67 ± 0.13	0.035 ± 0.03	–	0.63 ± 0.12	1.09 ± 0.18	2.56 ± 0.36
Si	28.65 ± 2.19	38.18 ± 1.86	21.65	16.72 ± 1.29	18.35 ± 1.54	5.36 ± 0.53
P	0.57 ± 0.10	–	0.66	0.36 ± 0.08	0.83 ± 0.12	2.37 ± 0.25
S	1.66 ± 0.17	–	1.70	1.35 ± 0.14	2.34 ± 0.22	4.52 ± 0.40
Cl	3.23 ± 0.25	2.12 ± 0.52	1.80	2.51 ± 0.21	3.56 ± 0.29	8.27 ± 0.64
K	12.16 ± 0.71	0.90 ± 0.28	15.26	15.09 ± 0.86	19.46 ± 1.18	26.22 ± 1.71
Ca	2.02 ± 0.17	0.31 ± 0.12	–	2.06 ± 0.17	3.20 ± 0.25	6.01 ± 0.43
N		0.66 ± 0.66	–			
Al		0.22 ± 0.22	–			

The XRD patterns of the ash samples obtained by calcination at 600 and 800 °C for 6 h (**Figure 6c**), red and blue lines, respectively) display sharp Bragg reflections attributed to the following inorganic compounds: silvite (KCl, cubic, pdf#41-1472) and arcanite (K_2_SO_4_, orthorhombic, pdf#05-0613), as major compounds, and magnesite (MgCO_3_, rhombohedral, pdf#08-0479) and hydroxylapatite (Ca_5_(PO_4_)_3_(OH), hexagonal, pdf#09-0432), in small quantities.

As Si did not form any crystalline compound during the calcination of the leaf at 600 °C or 800 °C, we performed a post-chemical treatment of the ash produced at 600 °C. The broad ill-defined diffraction peak centered at about 22° in the XRD pattern of the latter sample (**Figure 6c**, black line) is attributed to disordered silica (SiO_2_) (Li et al., 2020). Plants are known to absorb monosilic acid (H_4_SiO_4_) from the soil water which is taken up by roots and precipitated as hydrated amorphous silica (SiO_2_*nH_2_0) in various parts of the body. The accumulation of silica is implicated in several vital biological functions, such as structural support, as a cost-effective alternative to lignin, in defense against herbivores and pathogenic fungi or germs, for mechanical issues, and for the reduction of climatic and chemical stresses (Keutmann et al., 2015; Strömberg et al., 2016). The other diffraction peaks observed in the same XRD pattern (**Figure 6c**, black line) correspond to arcanite, magnesite and hidroxylapatite. The chemical treatment performed on the *A. donax* ash after calcination at 600 °C reduced dramatically the amount of silvite (KCl) (*cf.* reduction from 24.58% to 3.26% for K and 3.2% to 0.59% for Cl in **Table 1**). As expected, the FT-IR spectrum of the *A. donax* ash produced at 600 °C for 6 h and then treated chemically displayed an intense band at 1079 cm^-1^ and a shoulder at 1018 cm^-1^ (not shown), associated with the Si-O-Si stretching vibration.

It was obviously essential to further discriminate the composition of the organic compounds present in the cuticular waxes of adaxial and abaxial surfaces of the *A. donax* leaf. In general, waxes are mainly composed of primary and secondary alcohols, alkanes, triterpenoids, alkyl esters, β- diketones, aldehydes, alkylresorcinols, and fatty acids. The relative proportions of these compounds vary as a function of the species, vegetal organs, age or stage of development, and environmental stresses.

The most prevalent components of the cuticular surfaces of *A. donax* leaves are presented in **Table 3**. As explained in detail elsewhere by some of us (Rodríguez et al., 2021), to improve the accuracy of compounds’ identification, only those substances that provided a Match Factor higher than 800 were selected for data processing. In addition, blanks helped identify some procedural interferences, including glycerol, while ELSS helped recognizing cis-13-docosenamide, oleamide and tris(2,4-di-tert-butylphenyl) phosphate as procedural and system contaminants. Therefore, these substances, together with other minor phthalates (likely derived from materials used during cuticular waxes extraction) were not considered in total area calculations. Based on the above criteria, fourteen substances belonging to five main classes (alcohols, aldehydes, esters, fatty acids and triterpenoids) were identified in the cuticular waxes of *A. donax* leaves (**Table 3** and **Table S2** in the **Supplementary Material**).

**Table 3:** Relative amounts (%, mean + standard deviation) of the most abundant compounds identified in the *A. donax* leaf cuticular waxes.

Compound	Chain length	Log	Adaxial		Abaxial	
			Epicuticular	Intracuticular	Epicuticular	Intracuticular
1-octacosanol	C_28_	12.6	2.7 ± 0.9	n.d.	5.4 ± 1.3	3.5 ± 1.8
1-triacontanol	C_30_	13.6	12.5 ± 2.7	16.2 ± 3.4	23.9 ± 9.7	15.4 ± 2.5
1-dotriacontanol	C_32_	14.6	3.8 ± 1.0	5.6 ± 1.6	4.6 ± 1.4	3.1 ± 0.7
**Total alcohols**			**27.5 ± 2.9^BC^**	**30.0 ± 3.7^AB^**	**31.8 ± 5.5^A^**	**24.9 ± 4.8^C^**
monopalmitin^a^	C_19_	5.6	1.9 ± 0.2	1.8 ± 0.1	n.d.	1.3 ± 0.0
octadecyl acetate	C_20_	8.7	8.0 ± 1.1	7.7 ± 0.9	2.4 ± 0.6	2.6 ± 0.6
eicosyl acetate	C_22_	9.7	4.2 ± 0.6	4.1 ± 0.4	n.d.	1.7 ± 0.1
**Total esters**			**14.0 ± 1.5^A^**	**13.6 ± 1.2^A^**	**2.4 ± 0.6^B^**	**3.6 ± 1.5^B^**
stigmasterol	C_29_	9.4	5.6 ± 0.9	6.8 ± 1.3	2.8 ± 1.0	3.3 ± 0.6
lupeol	C_30_	9.2	n.d.	n.d.	15.9 ± 8.5	20.6 ± 1.9
β-amyrin	C_30_	9.2	42.3 ± 4.0	38.5 ± 3.1	25.1 ± 8.7	32.2 ± 3.9
**Total triterpenoids**			**48.7 ± 3.7^C^**	**45.3 ± 3.1^C^**	**57.5 ± 6.1^B^**	**62.7 ± 4.4^A^**
palmitic acid	C_16_	7.2	4.6 ± 1.5	4.4 ± 1.2	2.3 ± 0.7	2.4 ± 0.4
triacontanal	C_30_	13.6	5.6 ± 1.8	7.0 ± 2.0	6.6 ± 1.1	7.2 ± 0.8

Different letters in a row correspond to statistically significant (p<0.05) differences between means of major chemical classes.

n.d., not detected.

a Sum of both positional isomers.

b Predicted data generated using the US Environmental Protection Agency’s EPISuite™.

**Table 3** clearly highlights the predominance of triterpenoids in both surfaces of the *A. donax* leaf, followed by alcohols, esters and other trace constituents. The adaxial surface is characterized by a high content of triterpenoid alcohols, ranging from 38.9 to 54.4%, of which β-amyrin represents up to 92.0% of this chemical class. The pentacyclic β-amyrin is a triterpenoid saponin which may play a role to maintain flexibility in the cuticle and avoid cuticular cracks (Faizal and Geelen, 2013). In our study, the adaxial surface revealed slight, but significantly lower content of total triterpenoids in comparison to the abaxial surface (**Table 3**). Besides the prevalence of triterpenic compounds, aliphatic alcohols (mainly 1-triacontanol) also have a reasonable share of total cuticular waxes (22.8 - 35.1%), followed by esters (11.3 - 15.3%). No statistical differences were verified between the wax composition of the epicuticular and intracuticular surfaces.

A similar compositional pattern was observed in the abaxial side of *A. donax* leaf, i.e., high content of triterpenic alcohols, followed by aliphatic alcohols, but then this surface showed a much lower content of total esters and palmitic acid (*p* < 0.05, **Table 3**). It was reported that, while the presence of fatty alcohols is mandatory for wax crystal formation, the absence of esters does not affect epicuticular wax (Rowland and Domergue, 2012). Moreover, the intracuticular surface presented a lower relative percentage of total alcohols in comparison to the epicuticular surface.

To the best of our knowledge, there are only two studies in the literature regarding the composition of the cuticular waxes of *A. donax* leaves. Maffei et al. (Maffei, 1996) solely focused on the alkane content and were able to detect vestigial amounts of C_23_ to C_36_ alkanes of which nonacosane was the most predominant one. In our study, we were able to confirm the occurrence of nonacosane and heptacosane, but only in the case of the total extract obtained after chloroform immersion. On the other hand, Chaudhuri and Ghosal (Chaudhuri and Ghosal, 1970) reported triacontane (C_30_) as the major alkane found in leaves extracted with petroleum ether (60-80°). In addition, these authors also pointed out 1-triacontanol as the major component of n-alkanols, as well as the presence of triterpenes, including both α- and β-amyrin and stigmasterol (Chaudhuri and Ghosal, 1970).

Coelho et al. (Coelho et al., 2007) analysed the chloroformic extract of *A. donax* reed stems (including nodes and internodes) and provided a detailed description of the chemical composition of lipophilic extractives. They found high amounts of C_14_–C_32_ fatty acids (mostly palmitic acid, C_16_), followed by triterpenols (mainly β-sitosterol), esters (mostly monopalmitin) and other minor lipid classes. Recently, Pansuksan et al. (Pansuksan et al., 2020) characterized the phytochemical constituents *in A. donax* rhizome extracts obtained after sequential extraction, based on the polarity index of various solvents. The rhizome is composed of high amounts of β-sitosterol, followed by β-stigmasterol, (Z,Z)-9,12-octadecadienoic acid, palmitic acid, 2-monopalmitin and several minor hydrocarbons. Soler et al. (Soler et al., 1983) provided data regarding the epicuticular seed wax of *A. donax* and, similarly to our study, nonacosane, monopalmitin, 1-triacontanol, and palmitic acid were some of the most prevalent substances that emerged amongst their chemical classes. Hence, despite the lack of information on the cuticular waxes composition in *A. donax* leaves, some identical trends are confirmed between different plant sections.

Cuticular waxes of each section showed relative compositional variability, leading to distinct hydrophobic surface behaviour. According to the magnitude of the octanol/water partition coefficient (K_OW_) and the relative composition of the *A. donax* leaves (**Table 3**), the epicuticular abaxial surface demonstrates higher hydrophobicity (K_OW_ ≈ 8.8) likely owing to its higher relative content of 1-triacontanol and β-amyrin. It is followed by the intracuticular abaxial surface (K_OW_ ≈ 8.6), the intracuticular adaxial surface (K_OW_ ≈ 8.4) and finally by the epicuticular adaxial surface (K_OW_ ≈ 8.2).

**Figure 7:**
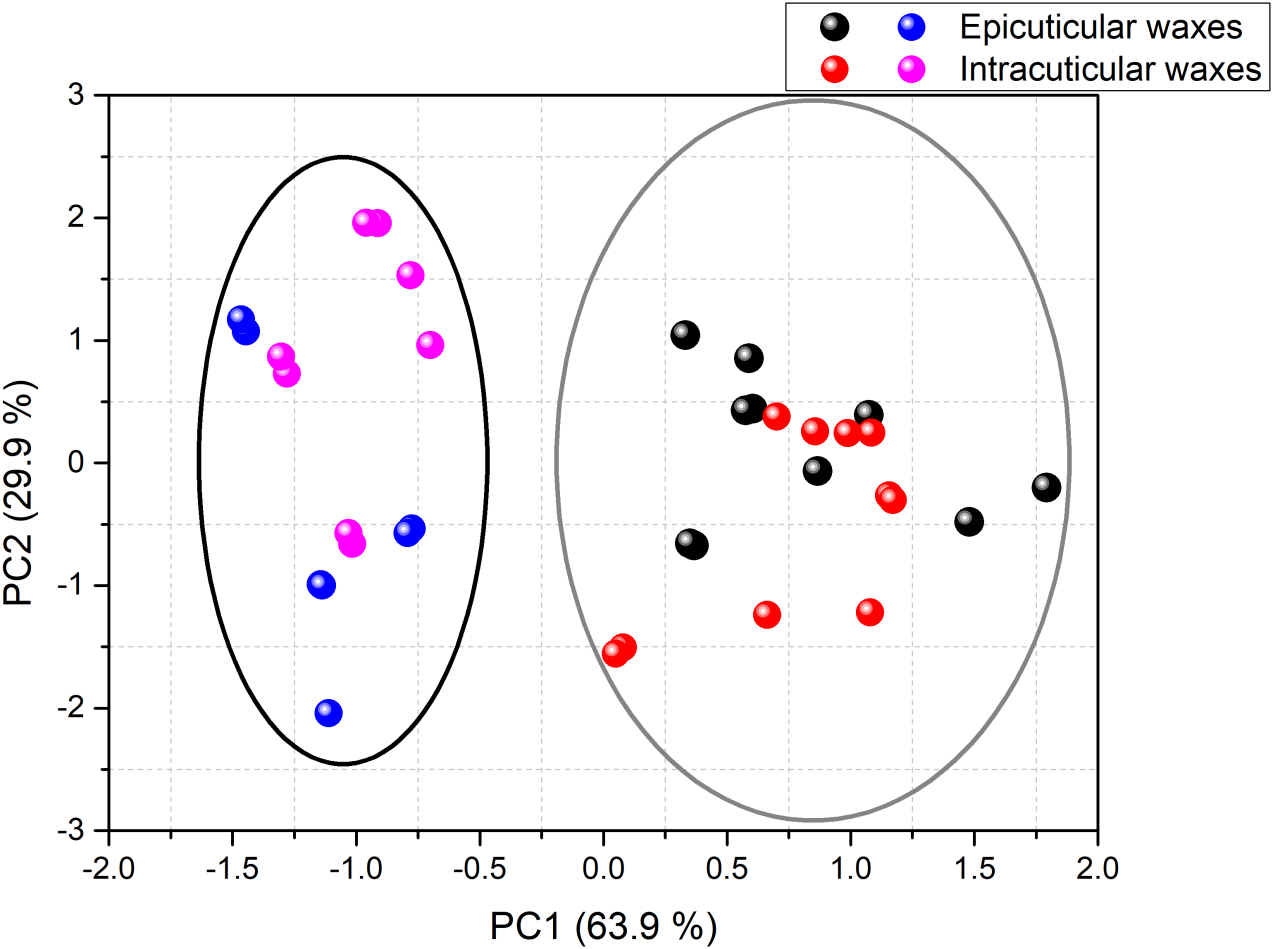
PCA of the composition of the cuticular waxes of green mature *A. donax* leaves: adaxial epicuticular (black symbols), adaxial intracuticular (red symbols), abaxial epicuticular (blue symbols) and abaxial intracuticular (pink symbols). The PC1 factor discriminated the adaxial surface (grey elipse) from the abaxial surface (black elipse).

A PCA test was conducted to classify different surfaces based on their overall waxes’ composition. This analysis allowed explaining 93.8% of the total data variance by using two principal components, as represented in **Figure 7**, whose variable communalities were all higher than 0.866. Herein, two distinct groups were identified that correspond to the adaxial and abaxial surface. The first principal component (PC1) factor, which comprises 63.9% of the total variance, can discriminate the adaxial surface located in the positive region (**Figure 7**, grey elipse) from the abaxial surface in the negative region (**Figure 7**, black elipse). This finding may be correlated with the fact that the adaxial surface was characterized by a higher content of esters (PC1 loading = 0.961) and palmitic acid (PC1 loading = 0.944), whereas the abaxial surface showed greater content of triterpenoids (PC1 loading = -0.714). The second principal component (PC2) factor, which accounts for 29.9% of the total variance observed, was able to slightly discriminate the intracuticular abaxial from the remaining surfaces likely due to its lower relative percentage of total alcohols (PC2 loading = -1.008).

Barthlott et al. (Barthlott et al., 1998) and Ensikat et al. (Ensikat et al., 2000) noted that the occurrence of platelet crystals may be correlated with the dominance of primary alcohols or triterpenoids. They also linked the presence of rodlet crystals with the dominance of triterpenoids, esters, primary alcohols, polymeric aldehydes, or ketones. Thus, the components detected by chromatographic analysis in the extracted waxes of the *A. donax* leaf are in perfect agreement with the micromorphology of the epicuticular waxes observed by SEM (**Figure 5**).

**Figure 8:**
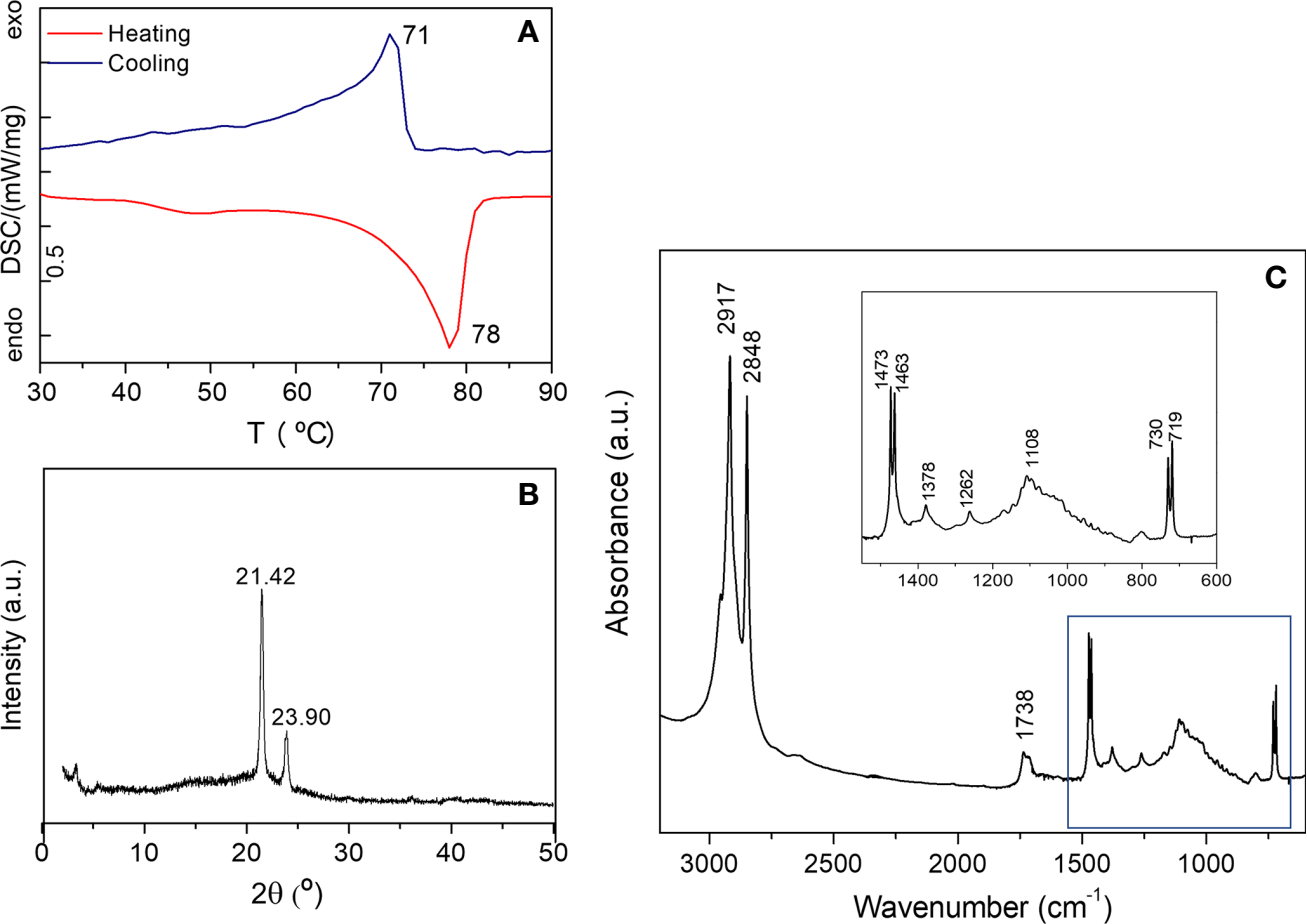
DSC curve **(a)**, XRD pattern **(b)** and FT-IR spectrum **(c)** of the cuticular waxes extracted from the green mature *A donax* leaf.

The DSC thermogram of the waxes extracted from the *A. donax* leaf are displayed in **Figure 8A**. The DSC curve of the waxes corresponding to the heating cycle displays two endothermic peaks. While the strong event peaking at higher temperature (78°C) can be assigned to the melting of the wax (total enthalpy of 115.1 J/g), the very weak and broad one around 40-50°C is assigned to a solid-solid transition. It may be due to an orthorhombic to hexagonal structural transition (Basson and Reynhardt, 1991; Reynhardt and Riederer, 1994) and very likely corresponds to hydrocarbons with chain lengths ranging from 21 to 39 carbon atoms (Nasri et al., 2014). The extracted *A. donax* waxes were of relatively high purity, as evidenced by the presence of a single sharp and nearly symmetrical melting peak. The recrystallization peak is centered at 71°C (**Figure 8A**).

In the XRD pattern of the waxes extracted from a green mature *A. donax* leaf only two peaks are visible at 21.42 and 23.90° (**Figure 8b**), revealing the formation of an orthorhombic crystal lattice (Reynhardt and Riederer, 1994). A weak peak around 3.24 ° of unknown nature, is also detected. Using the Bragg equation, *nλ* = 2*dsen*(*θ*), where is the diffraction order (natural number), λ the wavelength of used radiation, and θ the angle of the incoming radiation to the crystal lattice, a structural unit distance *d* = 4.15 Å was deduced.

The FT-IR spectrum of the *A. donax* wax represented in **Figure 8c** exhibits two intense bands at 2917 and 2848 cm^−1^ attributed to the asymmetric and symmetric stretching of aliphatic hydrocarbons, respectively. The in-plane vibrations of aliphatic hydrocarbons are located at 1470 and 1378 cm^−1^, while the band due to the rocking of the same vibrational group is found at 720 cm^−1^ (Svečnjak et al., 2015; Rodriguez-Saona et al., 2017). The pair of bands at 719 and 730 cm^−1^ and that at 1470 cm^−1^ (1473 and 1463 cm^−1^) are a fingerprint of an orthorhombic lattice (Chapman, 1955; Bernays et al., 1976). In addition, two medium absorption bands can be seen at 1738 cm^−1^ (shoulder at 1710 cm^−1^) and 1174 cm^−1^. The latter events are clearly related to the presence of carboxyl groups of fatty acids, triterpenoids, aldehydes and esters, the compounds detected by chromatographic analysis in the extracted waxes.

### 3.5 Surface wettability

Wetting is defined as the ability of a liquid to maintain contact with a solid surface, resulting from intermolecular interactions when the two are brought together. The degree of wetting is determined by a force balance between adhesive and cohesive forces, which is often characterized by the CA (Wang et al., 2015). In the case of the surface of plant leaves, the (super)hydrophobicity relies on the combination of the chemical composition of the epicuticular waxes (often hydrophobic 3D wax crystalloids with hierarchical micro- and nanostructures (Barthlott et al., 2017). The main factors that affect leaf wettability include the content and microstructure of the epidermal wax, the number, size and pattern of trichomes, the stomatal density, the shape of epidermal cells, and the leaf water status. Typically, the WCA of a leaf increases with the increase of the wax content.

**Figure 9:**
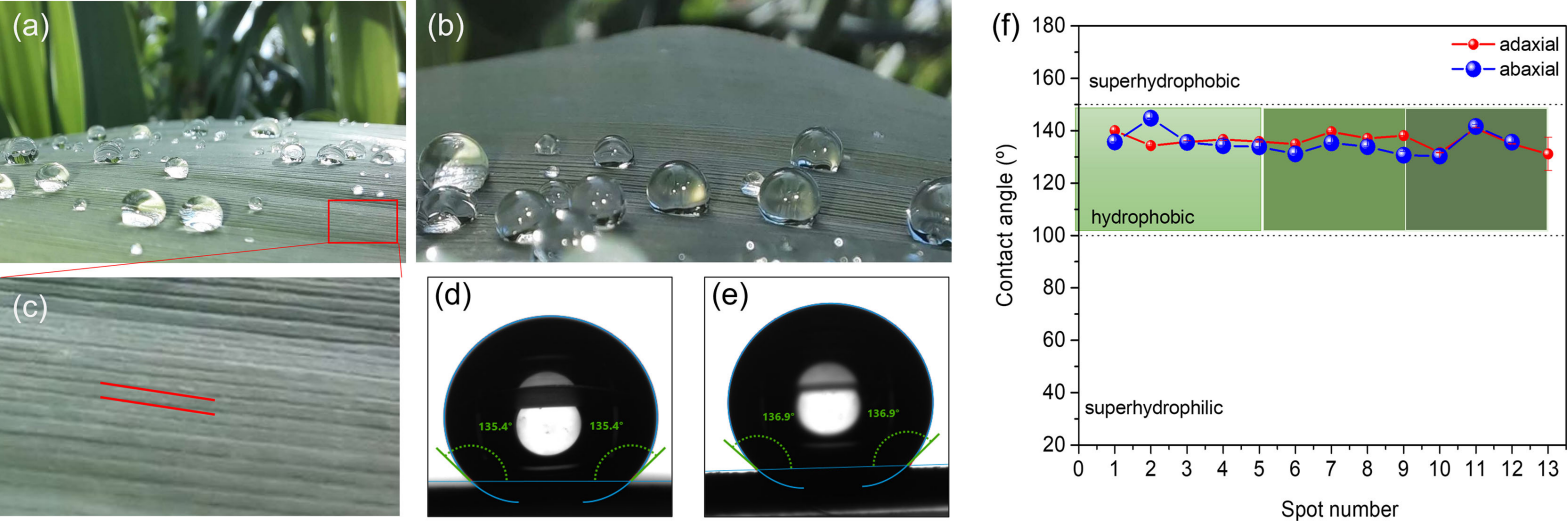
Green mature *A. donax* leaf: View of drops of water sitting on the abaxial surface **(a,b)**; magnification of the abaxial surface showing parallel vein directions (red lines) along which the WCAs were measured; **(c)** WCA of the adaxial surface; **(d)** WCA of the abaxial surface **(e)**. Static WCA values of the adaxial (blue symbols) and abaxial (red symbols) surfaces **(c)** of green mature *A. donax* leaf **(f)**.

**Figures 9a, b** show spherical water droplets sitting on the abaxial surface of a green mature A. *donax* leaf. The static WCAs were measured along parallel vein directions of leaf samples, as indicated by the red lines in **Figure 9c**. The WCA values of the adaxial and abaxial surfaces are represented in **Figures 9d, e**, respectively. The plot of **Figure 9f** reproduces the WCA values of the adaxial (blue symbols) and abaxial (red symbols) surfaces. The static WCAs of the adaxial and abaxial surfaces of green mature, but also juvenile, A. *donax* leaves are collected in **Table 4**. The magnitude of the WCA values points out that the *A. donax* leaf surfaces are both hydrophobic (**Figure 9f**) and **Table 4**), showing average WCA values of 135.2± 4.° and 136.2± 2.8° in the case of the adaxial and abaxial green mature leaves, respectively. The WCA of a juvenile *A. donax* leaf was found to be considerably lower (**Table 4** and **Figure S2** of **Supplementary Material**).

**Table 4:** Static water contact angles (in °) of the adaxial and abaxial surfaces of green mature and juvenile *A. donax* leaves.

Leaf		Section 1		Section 2		Section 3	
		Average	Highest	Average	Highest	Average	Highest
green mature	adaxial	135.5 ± 4.0	144.9	132.7 ± 1.9	135.3	136.3 ± 3.8	140.9
	abaxial	136.5 ± 2.0	140.2	137.4 ± 1.7	139.6	134.7± 3.9	141.1
juvenile	adaxial	112.7 ± 12.6	135.4	115.2 ± 4.5	122.6	106.3 ± 14.4	131.3
	abaxial	110.6 ± 11.7	125.2	110.0 ± 9.8	127.6	86.6 ± 3.7	92.9

### 3.6 Optical properties

**Figure 10:**
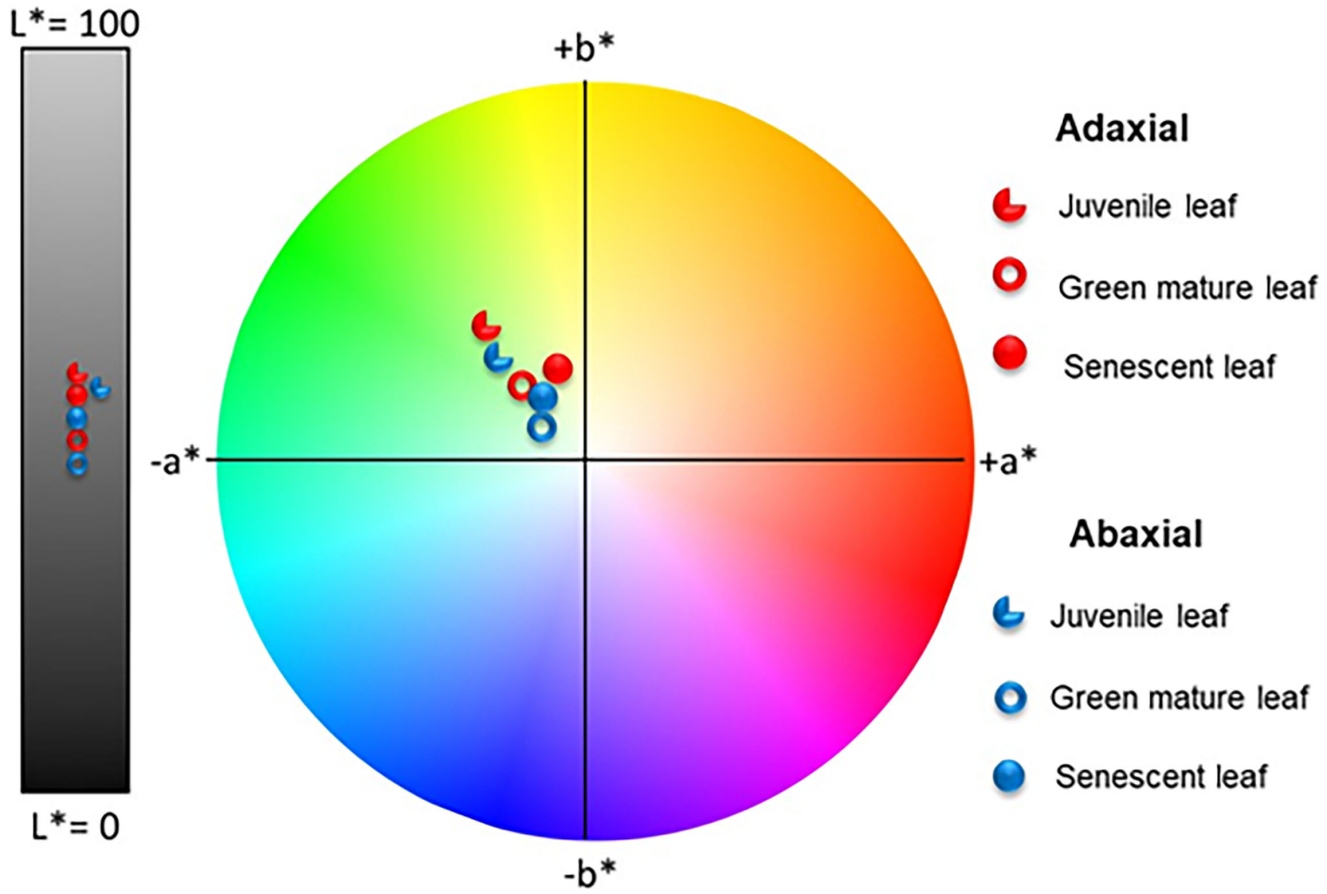
Color coordinates of the adaxial (red symbols) and abaxial (blue symbols) surfaces of juvenile, green mature, and senescent leaves of *A. donax* in the CIEL *a*b* color space diagram.

The quantification of the color exhibited by the abaxial and adaxial surfaces of juvenile, green mature, and senescent *A. donax* leaves was analyzed using the CIEL*a*b* color chromaticity diagram. The results of these measurements are reproduced in **Figure 10**.

The average values for the coordinate L* of the adaxial surfaces of juvenile, green mature, and senescent leaves of *A. donax* were always higher (i.e., the surfaces were brighter) than those of the corresponding abaxial surfaces independently of the plant development stage. In contrast the average values for coordinate a* changed, as expected, with time, shifting to less negative values (the leaf became less green) in the order juvenile < green mature < senescent. Concerning coordinate b*, its value first dropped (the leaf became less yellow) and then increased (the leaf became more yellow).

**Figure 11:**
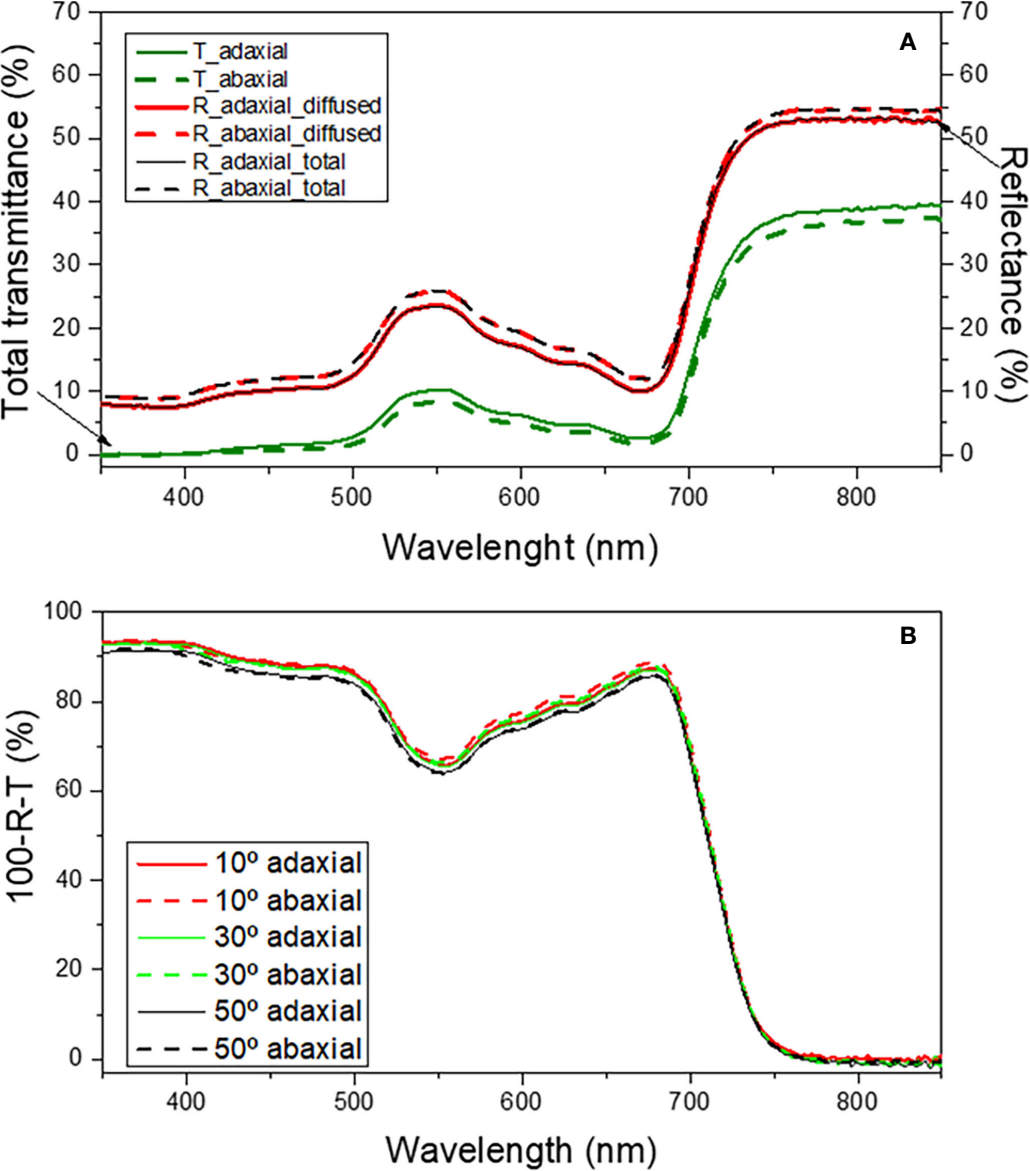
Total transmittance (T, green lines), diffuse reflectance (R, red lines) and total reflectance (R, black lines) **(a)** and total absorbance as a function of incident angle **(b)** for the adaxial (solid lines) and abaxial (dashed lines) surfaces of the green mature *A donax* L. leaf in the 350-850 nm spectral region.

**Figure 11A** shows the total transmittance (T) spectra together with the total and diffused reflectance (R) spectra in the wavelength range of 350-850 nm for the light incident on the adaxial and abaxial surfaces of green mature *A. donax* leaves.

The total reflectance from the adaxial and abaxial surfaces (**Figure 11A**, black solid and dashed lines, respectively) is practically identical in most part of the spectral region examined. Moreover, the diffused reflectance (**Figure 11A**, red solid and dashed lines, respectively) dominates the total reflectance indicating that the specular reflectance is negligible and hence explaining the lack of glossiness in the leaf surfaces. Expectedly, the total reflectance peaks (~25%) near 550 nm, the green region of the visible spectrum. Even though the reflectance is essentially the same on both surfaces, it may marginally vary (~1-2%) in some wavelength ranges. For instance, **Figure 11A**, black dashed and solid lines demonstrates that the total reflectance from the abaxial surface is slightly higher than that of the abaxial surface in the 350-450 nm interval, respectively.

The total transmittance through the adaxial (**Figure 11A**, green solid line) and abaxial (**Figure 11A**, green dashed line) surfaces differs very little, a situation analogous to that encountered for the reflectance features of the two surfaces. The similarity in the reflectance and transmittance characteristics of the adaxial and abaxial surfaces suggests that the absorption of incident light from the two surfaces must be comparable.

As expected, the results reveal that the absorbance varied only slightly when light was incident on the adaxial (**Figure 11b**, thick lines) or on the abaxial (**Figure 11b**, dashed lines) surfaces. Notably, for incidence angles ranging from 10-50°, the absorbance is nearly independent of the angle. In the 450-600 nm wavelength interval the variation of absorbance with changing incidence angles was ~1-2%.

The similarity in the optical responses exhibited by the adaxial and abaxial surfaces of the *A. donax* leaf when light is incident is a unique characteristic of this species that distinguishes it from most plant leaves. At this point it is useful to recall that, while the diffused reflectance, the dominant component of the total reflectance, originates from the scattering of light in the interior (mesophyll region) of a leaf, the specular reflectance, including polarization of the reflected light, is dictated by the air/cuticle interface and the morphology at the surface (Vogelmann, 1993). Therefore, the internal structure of a leaf is important to understand the differences in optical responses when light is incident on the adaxial or abaxial surfaces. It may be inferred from **Figure 3** that the morphology and arrangement of the mesophyll cells below the epidermis layers is fairly symmetrical with respect to the two surfaces of the leaf. This may account for the similar optical response from the adaxial and abaxial surfaces when light is incident. Although SEM findings provided clear evidences that the adaxial and the abaxial surfaces of the *A. donax* leaf differ markedly in terms of the epicuticular wax density (**Figure 5**), the influence of this effect on the total reflectance is negligible, as it affects the specular component more than the diffused component.

Based on the data retrieved from this study, we are led to conclude that the ecological success of the *A. donax* species along the Mediterranean basin and western Eurasia can be correlated with the easy growth of the plant, and its epidermic resistance. A response like this is in perfect agreement with the description reported by other authors for the *Poaceae* family (Prychid et al., 2003; Motomura et al., 2006). Moisture supplied by soil and the colonization of open habitats, under the effect of water streams, were suggested as environmental tolerances for *A. donax* (Quinn and Holt, 2017). The resistance of individuals might have been the main priority, and the low development of epicuticular waxes - and therefore the hydrophobicity - could be the main consequences of this chemophenetic strategy. The physical-chemical properties developed by the epicuticle along the various growth stages of the leaves also confirm this strategy: higher concentration of waxes for juvenile leaves, but higher water isolation for green mature leaves, in agreement with previous studies aimed at analyzing the tolerance of this species to dry conditions (Zegada-Lizarazu et al., 2018). The set of histological and physical-chemical data obtained in the present work for the epicuticle of the *A. donax* leaf, as well as the environmental continuity evidenced, demonstrate clear adaptations of this species to the environmental characteristics of its habitats. The lack of fertility for this species or its genetic homogeneity in the Mediterranean region (especially when compared with its Asiatic distribution), have been traditionally suggested to explain its potential invasiveness (Hardion et al., 2014b; Jiménez-Ruiz et al., 2021). However, this is not a consensual descriptor (Petit et al., 2004). The hypothesis of colonization from eastern Asia to the Mediterranean basin could be the consequence of a Plio-Pleistocene glacial-interglacial event (Fiz-Palacios et al., 2010; Carlson et al., 2012; Nieto Feliner, 2014; Martín-Hernanz et al., 2021). A continuous lineage process from eastern Eurasia to the Mediterranean basin was confirmed recently (Zecca et al., 2020). On the basis of this hypothesis, natural eastern-to-western migrations would give support to the expansion of *A. donax* along the Mediterranean basin and western Eurasia, and anthropic influence would be involved in the late stages of this process (Inda et al., 2014). In this regard, the epicuticular features observed for this taxon would have definitely played a pivotal role for the success of this colonization. The presence of siliceous structures, such as phytoliths and bulliform cells (Morris et al., 2009a; Morris et al., 2009b; Coe et al., 2013), or the predominance of triterpenoids as an allelopathic and toxicity mechanism, are considered decisive in ecological invasiveness (Kohli et al., 2006; Hussain et al., 2019). Nevertheless, additional information, namely archeological approaches, are required to classify this species as an archaeophyte across the Mediterranean basin (Lloret et al., 2011; Hardion et al., 2014b). In this context, archeological evidence pointed out the possibility of replacement of wild reeds (e.g., *Typha* spp., *Phragmites australis*) by *A. donax*, with very similar histological and physical-chemical characteristics, for anthropic purposes, like agriculture, construction, or others (Boulos and Fahmy, 2007; Balbo, 2015; Romano et al., 2021). The characterization of leaf epicuticle reported in the present work demonstrated undeniably the easy growth and adaptation of this species to arid and semi-arid habitats. In particular, the presence of relevant concentrations of siliceous structures along the leaf cuticle is known to be decisive to prevent water loss (Bibi et al., 2022), abiotic-biotic stresses, or for plant efficiency in general (Cooke and Leishman, 2011; Cooke and Leishman, 2016; Landi et al., 2017; Rao et al., 2017) and extremely useful for productive purposes (Corno et al., 2014; Webster et al., 2016). The Si-accumulation along the epicuticle of leaves that enhanced their strength and rigidity (Ma and Yamaji, 2006; Cooke and Leishman, 2011; Shakoor et al., 2014) is pivotal to understand the expansion of this species along the Mediterranean basin and western Europe, from western Asia, where it was traditionally used by human communities for the construction of utilities (Kouli, 2012; Spengler and Willcox, 2013; de Klerk and Joosten, 2021). The analysis of phytoliths in mono- and dicotyledonous epidermis has proved to be an effective tool for obtaining reliable quantitative information regarding plant-related activities, such as agriculture and farming (Delhon et al., 2020; Goude et al., 2020). Thus, our results confirm the ecological strategy of *A. donax*, suggesting recent colonization in the Mediterranean basin. In this sense, the genetic homogeneity observed for this species along this area and its vegetative reproductive strategies are also decisive to explain this ecological evolution (Haddadchi et al., 2013; Guarino et al., 2019). Under this perspective, *A. donax* could be considered a successful example of Neolithization (Langgut et al., 2021; Leppard, 2022).

## 4 Conclusions

In the light of an ecological perspective, the results derived from the present work suggest relevant adaptations of the *A. donax* leaf to arid and semi-arid environments. The set of information collected here highlights the remarkable robustness of the leaf epicuticle, arising from the presence of silica and cork cells, bulliform cell rows, and triterpenoid compounds. The striking similarity found between the adaxial and abaxial surfaces of *A. donax* leaf in terms of morphology of the epicuticular waxes, contact angle values, and optical response, plus the presence of silica phytoliths and bulliform cells, as well as the prevalence of triterpenoids on the surface of the leaves, confirm chemophenetic and histological adaptations. The remarkable cuticular resistance of the *A. donax* leaf seems to have played a key role on the neolithization of this species along the Mediterranean basin and surrounding regions.

## Data availability statement

The raw data supporting the conclusions of this article will be made available by the authors, without undue reservation.

## Author contributions

SN performed leaf collection and participated in leaf calcination, organized all data, was deeply involved in the discussion of the characterization data and wrote the first draft of the manuscript; AG participated in leaf calcination, recorded the SEM images, XRD patterns and registered the DSC curves, PN recorded the static contact angle data, POM images and the Commission Internationale d’Éclairage (CIE) L*a*b* color coordinates; MF recorded the static contact angle data and POM images; AM performed the epicuticular and intracuticular waxes extraction, and recorded the FTIR spectra, EB performed the morpho-anatomical and hystological characterization and participated in the discussion of the corresponding data; JR performed the analysis of the potential distribution and participated in the environmental characterization of the leaf; RC participated in the chemical characterization of the epicuticular and intracuticular waxes and corresponding discussion; AB participated in the chemical characterization of the epicuticular and intracuticular waxes, AR recorded the optical transmittance and reflectance curves and participated in the discussion of the resulting data; SC contributed to the discussion of the chemical characterization of the intra and intercuticular waxes; SA contributed to the discussion of the optical data, VZB was deeply involved in the discussion of the results and in the writing of the manuscript; AC participated in environmental characterization of leaf and was deeply involved in the discussion of the results and in the writing of the manuscript. All authors contributed to manuscript revision, read and approved the submitted version.

## Acknowledgments

This research was funded by National Funds from Foundation for Science and Technology (FCT) and FEDER through POCI-COMPETE 2020-Operational Programme Competitiveness and Internationalization in Axis I-Strengthening research, technological development and innovation (UIDB/00616/2020 (https://doi.org/10.54499/UIDB/00616/2020) and UIDP/00616/2020 (https://doi.org/10.54499/UIDP/00616/2020) to CQ-VR, UIDB/04033/2020 to CITAB, UIDB/00195/2020 (https://doi.org/10.54499/UIDB/00195/2020) to FibEnTech and UID/QUI/50006/2020 to LAQV/Requimte) and by the project PORPLANTSURF - Superhydrophobic films inspired by the surface of plant leaves and petals from Northern Portugal (POCI-01−0145-FEDER-029785), financed by the European Regional Development Fund (ERDF) through COMPETE 2020-Operational Program for Competitiveness and Internationalization (POCI) and FCT. SN acknowledges FCT for the Assistant Research contract (2020-00805.CEEIND, https://doi.org/10.54499/2020.00805.CEECIND/CP1625/CT0001) in the scope of the Scientific Employment Stimulus. The authors acknowledge Patrick Joel Pais for the preparation of the fresh leaf for SEM analysis. PN acknowledges CQ-VR/FCT for a PhD grant (UI/BD/151084/2021). AR and SA acknowledge partial support from the Swedish Energy Agency (grant no. 49227-1). MF acknowledges FCT-UTAD for the contract in the scope of Decreto-Lei 57/2016−Lei 57/2017.

## Conflict of interest

The authors declare that the research was conducted in the absence of any commercial or financial relationships that could be construed as a potential conflict of interest.

## Publisher’s note

All claims expressed in this article are solely those of the authors and do not necessarily represent those of their affiliated organizations, or those of the publisher, the editors and the reviewers. Any product that may be evaluated in this article, or claim that may be made by its manufacturer, is not guaranteed or endorsed by the publisher.
