# Corrigendum: Leaf surfaces and neolithization - the case of *Arundo donax L*

**DOI:** 10.3389/fpls.2024.1452859

**Published:** 2024-07-16

**Authors:** Sílvia C. Nunes, Ana P. Gomes, Paulo Nunes, Mariana Fernandes, Ana Maia, Eunice Bacelar, João Rocha, Rebeca Cruz, Aline Boatto, Ajith P. Ravishankar, Susana Casal, Srinivasan Anand, Verónica de Zea Bermudez, António L. Crespí

**Affiliations:** ^1^ Fib EnTech - Fiber Materials and Environmental Technologies, University of Beira Interior, Covilhã, Portugal; ^2^ Centro de Química Vila Real (CQ-VR), University of Trás-os-Montes e Alto Douro, Vila Real, Portugal; ^3^ Department of Chemistry, University of Trás-os-Montes e Alto Douro, Vila Real, Portugal; ^4^ Centre for the Research and Technology of Agro-Environmental and Biological Sciences (CITAB), Department of Biology and Environment, University of Trás-os-Montes e Alto Douro, Vila Real, Portugal; ^5^ Herbarium and Botanical Garden, University of Trás-os-Montes e Alto Douro, Vila Real, Portugal; ^6^ Associated Laboratory for Green Chemistry (LAQV) of the Network of Chemistry and Technology (REQUIMTE), Department of Chemical Sciences, Faculty of Pharmacy, Laboratory of Bromatology and Hydrology, University of Porto, Porto, Portugal; ^7^ Department of Applied Physics, School of Engineering Sciences, KTH Royal Institute of Technology, Albanova University Centre, Stockholm, Sweden

**Keywords:** *A. donax* L. leaf, environmental adaptation, wax composition, hydrophobicity, optical properties, neolithization, chemophenetics and inorganic composition

In the published article, there was an error in the **Acknowledgments** statement regarding CQ-VR. The section previously stated:

“This research was funded by National Funds from Foundation for Science and Technology (FCT) and FEDER through POCI-COMPETE 2020-Operational Programme Competitiveness and Internationalization in Axis I-Strengthening research, technological development and innovation (UID/QUI/00616/2013 and UID/QUI/00616/2019 to CQ-VR, UIDB/04033/2020 to CITAB, UIDB/00195/2020 to FibEnTech and UID/QUI/50006/2020 to LAQV/Requimte) and by the project PORPLANTSURF - Superhydrophobic films inspired by the surface of plant leaves and petals from Northern Portugal (POCI-01−0145-FEDER-029785), financed by the European Regional Development Fund (ERDF) through COMPETE 2020-Operational Program for Competitiveness and Internationalization (POCI) and FCT. SN acknowledges FCT for Assistant Research contract (2020-00805.CEEIND) in scope of Scientific Employment Stimulus. The authors acknowledge to Patrick Joel Pais for the preparation of the fresh leaf for SEM analysis. PN acknowledges CQ-VR/FCT for a PhD grant (UI/BD/151084/2021). AR and SA acknowledge partial support from the Swedish Energy Agency (grant no. 49227-1). MF acknowledges FCT-UTAD for the contract in the scope of Decreto-Lei 57/2016−Lei 57/2017.”

The corrected statement appears below:

“This research was funded by National Funds from Foundation for Science and Technology (FCT) and FEDER through POCI-COMPETE 2020-Operational Programme Competitiveness and Internationalization in Axis I-Strengthening research, technological development and innovation (UIDB/00616/2020 (https://doi.org/10.54499/UIDB/00616/2020) and UIDP/00616/2020 (https://doi.org/10.54499/UIDP/00616/2020) to CQ-VR, UIDB/04033/2020 to CITAB, UIDB/00195/2020 (https://doi.org/10.54499/UIDB/00195/2020) to FibEnTech and UID/QUI/50006/2020 to LAQV/Requimte) and by the project PORPLANTSURF - Superhydrophobic films inspired by the surface of plant leaves and petals from Northern Portugal (POCI-01−0145-FEDER-029785), financed by the European Regional Development Fund (ERDF) through COMPETE 2020-Operational Program for Competitiveness and Internationalization (POCI) and FCT. SN acknowledges FCT for the Assistant Research contract (2020-00805.CEEIND, https://doi.org/10.54499/2020.00805.CEECIND/CP1625/CT0001) in the scope of the Scientific Employment Stimulus. The authors acknowledge Patrick Joel Pais for the preparation of the fresh leaf for SEM analysis. PN acknowledges CQ-VR/FCT for a PhD grant (UI/BD/151084/2021). AR and SA acknowledge partial support from the Swedish Energy Agency (grant no. 49227-1). MF acknowledges FCT-UTAD for the contract in the scope of Decreto-Lei 57/2016−Lei 57/2017”.

The authors apologize for this error and state that this does not change the scientific conclusions of the article in any way. The original article has been updated.

