# Supplementary material for: Leaf surfaces and neolithization - the case of Arundo donax L
Source: Front Plant Sci. 2022 Oct 5;13:999252. doi: 10.3389/fpls.2022.999252 (PMC9581231; doi:10.3389/fpls.2022.999252)
Supplement: Supplementary file 1 [file Table_1.docx]

**Supplementary Table 1.** Relation of the most discriminant environmental variables for the biogeographic and haplotypic strategies adopted by *A. donax* leaf.

|  | **Willks´Lambda** | **exact F** | **Significance** |
| --- | --- | --- | --- |
| p11 | 0.206 | 44.068 | 0 |
| bio14 | 0.161 | 40.679 | 0 |
| Tm4 | 0.139 | 36.310 | 0 |
| bio10 | 0.201 | 33.461 | 0 |
| bio3 | 0.122 | 33.121 | 0 |
| p4 | 0.112 | 30.146 | 0 |
| bio18 | 0.539 | 20.091 | 0 |
| Alt | 0.449 | 18.052 | 0 |

Note: p11 - average precipitation in November; bio14 - precipitation of the driest month, Tm4 - maximum temperature in April, bio10 - mean temperature of warmest quarter; bio3 - isothermality (bio2/bio7) (×100); p4 – average precipitation in April; bio18 - precipitation of warmest quarter; Alt – altitude.

The very low Willks´Lambda values, or exact F values found for the variables reveal no significant discrimination between Mb, T1 and T2. In this context, very high correlation between them is expected, as confirmed by PCA.
