# Supplementary material for: Leaf surfaces and neolithization - the case of Arundo donax L
Source: Front Plant Sci. 2022 Oct 5;13:999252. doi: 10.3389/fpls.2022.999252 (PMC9581231; doi:10.3389/fpls.2022.999252)
Supplement: Supplementary file 2 [file Table_2.docx]

| **Supplementary Table 2.** Chromatographic information regarding the most abundant compounds identified in different surfaces of *A. donax* leaf cuticular waxes | | | | | | |
| --- | --- | --- | --- | --- | --- | --- |
|  | **RETENTION INDEX** | **MATCH** | **REVERSED MATCH** | **MASS FRAGMENTATION PATTERN (*m/z*)** | | |
| Palmitic acid, TMS derivative* | 2050 | 917 | 930 | 73 | 117 | **313** |
| 1-Octadecanol, TMS derivative | 2050 | 917 | 930 | 75 | **327** | 328 |
| Octadecyl acetate | 2152 | 882 | 916 | **43** | 55 | 97 |
| Eicosyl acetate | 2208 | 883 | 906 | **43** | 83 | 97 |
| 1-Monopalmitin, TMS derivative* | 2607 | 880 | 935 | 73 | 147 | **371** |
| 1-Hexacosanol, TMS derivative* | 2940 | 840 | 870 | 57 | 75 | **439** |
| 1-Octacosanol, TMS derivative* | 3138 | 928 | 929 | 57 | 75 | **467** |
| Triacontanal* | 3251 | 807 | 942 | **43** | 57 | 82 |
| Stigmasterol, TMS derivative* | 3274 | 889 | 908 | 55 | **83** | 129 |
| β-Amyrin, TMS derivative | 3348 | 918 | 935 | 73 | 203 | **218** |
| 1-Triacontanol, TMS derivative* | 3334 | 922 | 922 | 57 | 75 | **239** |
| α-Amyrin, TMS derivative | 3406 | 900 | 914 | 73 | 189 | **218** |
| Lupeol, TMS derivative | 3435 | 922 | 923 | 68 | **73** | 129 |
| 1-Dotriacontanol, TMS derivative | 3530 | 878 | 879 | 109 | **189** | 190 |
| *Compounds whose retention times and *m/z* spectra were compared to those of reference standards. | | | | | | |
| Bold mass fragments indicate base peaks. | | | | | | |
