# Supplementary figures and images for: Leaf surfaces and neolithization - the case of Arundo donax L
Source: Front Plant Sci. 2022 Oct 5;13:999252. doi: 10.3389/fpls.2022.999252 (PMC9581231; doi:10.3389/fpls.2022.999252)

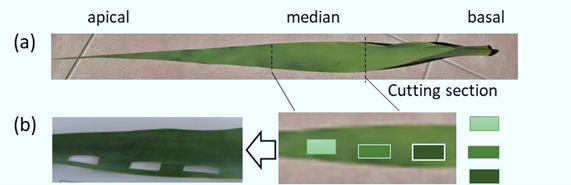

Supplement: Supplementary file 3 [file Image_1.tif]

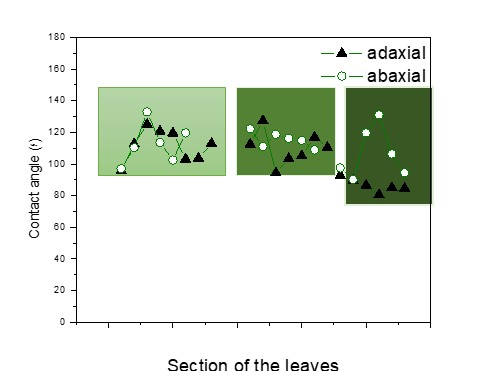

Supplement: Supplementary file 4 [file Image_2.tif]
